# Identification of soil type in Pakistan using remote sensing and machine learning

**DOI:** 10.7717/peerj-cs.1109

**Published:** 2022-10-03

**Authors:** Yasin Ul Haq, Muhammad Shahbaz, HM Shahzad Asif, Ali Al-Laith, Wesam Alsabban, Muhammad Haris Aziz

**Affiliations:** 1 Department of Computer Science and Engineering, University of Engineering and Technology Lahore Narowal Campus, Narowal, Pakistan; 2 Department of Computer Engineering, University of Engineering and Technology Lahore, Lahore, Punjab, Pakistan; 3 Department of Computer Science, University of Engineering and Technology Lahore, Lahore, Kala Shah Kaku, Punjab, Pakistan; 4 Computer Science Department, Copenhagen University, Copenhagen, Denmark; 5 Information Systems Department, Faculty of computer and Information Systems, Umm Al-Qura University, Makkah, Saudi Arabia; 6 College of Engineering & Technology, University of Sargodha, Sargodha, Sargodha, Pakistan

**Keywords:** Remote sensing, Spectral signatures, Digital soil mapping, Soil type, Random forest

## Abstract

Soil study plays a significant role in the cultivation of crops. To increase the productivity of any crop, one must know the soil type and properties of that soil. The conventional soil type identification, grid sampling and hydrometer method require expert intervention, more time and extensive laboratory experimentation. Digital soil mapping, while applying remote sensing, offers soil type information and has rapidity, low cost, and spatial resolution advantages. This study proposes a model to identify the soil type using remote sensing data. Spectral data of the Upper Indus Plain of Pakistan Pothwar region and Doabs were acquired using fifteen Landsat eight images dated between June 2020 to August 2020. Bare soil images were obtained to identify the soil type classes Silt Loam, Loam, Sandy Loam, Silty Clay Loam and Clay Loam. Spectral data of band values, reflectance band values, corrective reflectance band values and vegetation indices are practiced studying the reflectance factor of soil type. Regarding multi-class classification, Random Forest and Support Vector Machine are two popular techniques used in the research community. In the present work, we used these two techniques aided with Logistic Model Tree with 10-fold cross-validation. The classification with the best performance is achieved using the spectral data, with an overall accuracy of 86.61% and 84.41% for the Random Forest and Logistic Model Tree classification, respectively. These results may be applied for crop cultivation in specific areas and assist decision-makers in better agricultural planning.

## Introduction

Agriculture is the backbone of Pakistan’s economy, which heavily depends on its cash crops. In Pakistan, there are two main seasons for growing crops: Rabi (winter) and Kharif (summer). Crops of Rabi are sown in the month of October–November and Kharif in the months of April–May. Cash crops such as cotton, rice, and sugarcane are sown in May and harvested in November, while the crop of wheat is sown in November, and it becomes ready at the end of April (Rehman et al., 2015). Rich soil, timely irrigation and plowing are the necessities to achieve maximum yield. The maintenance of soil plays a crucial role in the production of food. A soil with better health will yield a better quality product which is more advantageous to humans. To obtain healthy food, it is necessary to maintain the fertility and health of the soil (Gomez & Lagacherie, 2016).

The nature of the plant environment depends on the soil quality, and it determines how much human life can flourish on earth (Crouse, 2018). Soil fertility comprises three main components, which are physical, chemical, and biological (Zeng et al., 2009). Soil fertility is the product of the soil’s inherent characteristics and the relationships between these three components. As the human population expands, the food requirement also increases, which harms the soil and the ecosystem (Foley et al., 2005). The growing population is causing the merger of small villages and farmlands into larger cities, making it necessary to improve per unit yield and exploration of new areas which can be used for cultivation. The most important thing for crop cultivation is to know about the properties and requirements of crop and land, *e.g*., soil type of that area, the chemical composition of the soil, available nutrients and minerals and weather properties in that region. Using uncultivated areas for farming requires their assessment to identify their suitability for some specific crops based on weather and irrigation options. The chemical and physical characteristic of soil is declining, causing soil debasement (Hengl et al., 2015; Stevens, 2015). For proper land usage, it is important to take into consideration the land dispersal, chemical and physical properties of soil on the surface of the earth (Feizizadeh & Blaschke, 2013). Experts require manual sample collection of these areas to identify and categorize these lands, which may involve expensive and time-consuming lab experiments.

Conventional soil texture identification methods have primarily depended on field surveys. The conventional way of soil type identification is soil sampling which requires expert intervention and extensive laboratory experimentation. Traditional ground-based surveys are reported to be expensive and time-consuming due to soil specimen and laboratory analysis, mainly when we are interested in identifying the soil type at a large scale, like the national or regional level (Behrens & Scholten, 2006; Dobos et al., 2001; Mulder et al., 2011). Therefore, traditional soil mapping approaches are not suitable for small farmers and assessment of land on a large scale (Basso et al., 2011). New methods for obtaining high-resolution soil texture are being established to overcome these issues. In Digital Soil Mapping (DSM), we identify the soil texture using secondary (non-soil) data sources to overcome the restrictions of conventional techniques and improve spatial coverage and detail of soil databases (Nanni & Demattê, 2006; Summers et al., 2011). DSM is not only cost-effective; it permits ascertainment of a purpose quantitative measure of forecast apprehension, which is generally not offered in conventional techniques (Hussain et al.). Remote sensing (RS) data promises secondary data sources to improve the digital soil map over the past few decades (Haldar et al., 2022). Using RS techniques rather than traditional methods to get these data can be time taking and painstaking (Stevens, 2015). These techniques do not devastate the sample or chemical deposits, and these are more efficient and not pricey (Palacios-Orueta & Ustin, 1998).

RS is a valuable technique for describing the spatial, spectral, and temporal properties noted from earth surface characteristics for spatially separated variables (Kneubühler et al., 2014; Pande et al., 2018; Pande et al., 2020). It is a technique of acquiring data through earth observation satellites. These satellites employ sensors that acquire reflectance from the earth’s surface in frequency bands known as frequency spectrums. The reflectance is recorded using active as well as passive sensors. Active sensors emit light through a laser-based system and record its reflectance providing information about earth terrain, whereas passive sensors, which are most common, use sunlight reflected from the soil surface (Bagheri, 2020; Hengl et al., 2015). Near infrared reflectance analysis (NIRA) is used to compute the land characteristics in shortwave and near infrared (Ben-Gera, 1968). The reflectance is recorded into different spectra, which provides a hyperspectral image that can be used to extract spectral signatures (Mohamed et al., 2018). It helped to find and validate the relationship between chemical data and spectral data using prediction equations on analysis of multiple linear regression (MLR). In a laboratory environment (Ben-Dor, Irons & Epema, 1999) applied this technique to the visible region to predict specific surface area, carbonate (CaCO_3_) contents, residual loss-on-ignition and total silica (SiO_2_) by simulating the bands of the Landsat satellite.

Remotely sensed images provide information on different visible and invisible bands, which is then used to calculate indices such as NDVI, Leaf area index (LAI), soil reflectance index, and other useful indices (Xue & Su, 2017). With high-resolution sensors and advanced technology, the bands obtained by remote sensing are increasing, thus helping us calculate better indices with more representation power (Khan et al., 2018). RS and spectroscopy are provide us with hyper spectral images (HSI). These images provide us with image texture information and spectral information. Image texture information depends on the relationship of neighboring pixels intensities and helps us classify soil type (Xue & Su, 2017). The spectral signature of a material is characterized by its form, the intensity of its reflectance and absorption bands which are electrical and vibrational transitions (Gomez & Lagacherie, 2016).

These spectral signatures are provided to machine learning algorithms, which learn the relationship of these indices with the target variable. These are automated computer algorithms and the provision of representative data is important for them to learn these relationships. There are three types of machine learning algorithms: supervised, unsupervised, and reinforcement learning (Mahesh, 2020). The choice of algorithms depends on your data types and what task you want to automate. Supervised learning algorithms are spoon-fed versions of machine learning as we label the data *corpus*, and the algorithm works with strict boundaries. This process is like “connecting the dots” because we select the type of information to feed the algorithm and we know the kind of desired results (Mahesh, 2020).

Soil texture comprises silt, clay, and sand contents and is a valuable property for many soil functions. Water retention, soil structure, aeration, capacity, susceptibility to erosion, cation exchange capacity, organic matter content, and others are swayed by soil texture, which is an important property in soil classification systems (Bousbih et al., 2019; Jia et al., 2017). In the selection and growth of crops, soil texture is one of the most important factors (Chakraborty & Mistri, 2015).

RS techniques for the estimation of soil texture have been used in many studies. In the laboratory, texture quantification spectral data models were created (Coleman, Agbu & Montgomery, 1993). Spectral standards attained in the laboratory help us recognize satellite standards; meanwhile, the perception of energy interaction matches what occurs in the laboratory, altering only the scale and configuration of the apparatus. Consequently, studies have been performed using laboratory data to simulate satellite data (Rehman et al., 2015). Studies (Hengl et al., 2015; Okparanma & Mouazen, 2013; Pham, Prakash & Bui, 2018) have showed the analytical capacity of the satellite data in relation to the proximal data.

Dynamic spatial panels of RS data and topographic, bioclimatic and soil conditions features are used to apply data-mining techniques. Xu, Wang & Chen (2018) proposed a spatio-temporal crop disease prediction framework based on ensemble learning techniques and spatio-temporal recurrent neural network (STRNN), which is an extension of recurrent neural networks (RNN) in time and space. For prediction precision analysis, they used a specific dataset based on reported cases of wheat yellow rust outbreaks in the Longnan city.

In recent years, Jia et al. (2017) used hyperspectral images to measure total soil nitrogen and classify the soil types. Spectral soil data emanating from the satellite are influenced by pixel size of images, atmospheric attenuations, soil cover, sensor spectral resolution, geometric view, and increased signal-to-noise ratio since variations are essential in the methods and models to enhance spectral responses. For instance, Liao et al. (2013) proposed a method for spatial estimation of soil surface texture using kriging, co-kriging and stepwise multiple regression and co-kriging gave them best prediction. da Silva Chagas et al. (2016) recently predicted the clay, silt, and sand contents, based on Landsat TM 5 bands and various indexes which not only helped them enhance accuracy of the models but also classify bare soil using the RF and MLR methods.

More recently, Demattê et al. (2018) proposed a quantification model for soil texture based on reflectance information from a range of bare soils acquired by intersecting multi-temporal satellite images. This model was applied to another region to calculate its pertinency. They used only bare soil spectral data collected using Landsat TM 7 satellite images of two distinct regions of Brazil. The model was trained using spectral data (acquired from six bands) and laboratory data (having size 0.00–0.20 m layer) of one region and extrapolated to another. Sandy loam, clayey loam, sandy, clayey, and very clayey soil are the differentiated textual classes.

Similarly, Forkuor et al. (2017) investigated six soil properties-silt, clay, sand, soil organic carbon, nitrogen, and cation exchange capacity of area 580 km^2^ situated in south-western Burkina Faso, by using the high-resolution satellite data (Landsat 8 and RapidEye), topography data and laboratory examined. They tested and compared four prediction models: random forest regression, support vector machine, stochastic gradient boosting, and multiple linear regression. Prediction of the model was validated on an independent set of similar soil samples (Forkuor et al., 2017).

In this context, the present study aims to identify the soil type by using the spectral signature of land through remote sensing data. This analysis is based on satellite measurements acquired over the Upper Indus Plain in Pakistan. We analyze the sensitivity of several indicators to soil texture and use the machine algorithms to determine soil type classifications. The objective of this study is to find out the answers to the following questions:

I. Is it possible to identify the soil type using remote sensing data?
II. How can we map spectral signature with soil type?
III. Which index calculated from remotely sensed images is more useful for soil type identification?
IV. Which machine learning algorithm is more effective at mapping the spectral signature to the relevant soil type?

## Methods

### Study site

**Figure 1:**
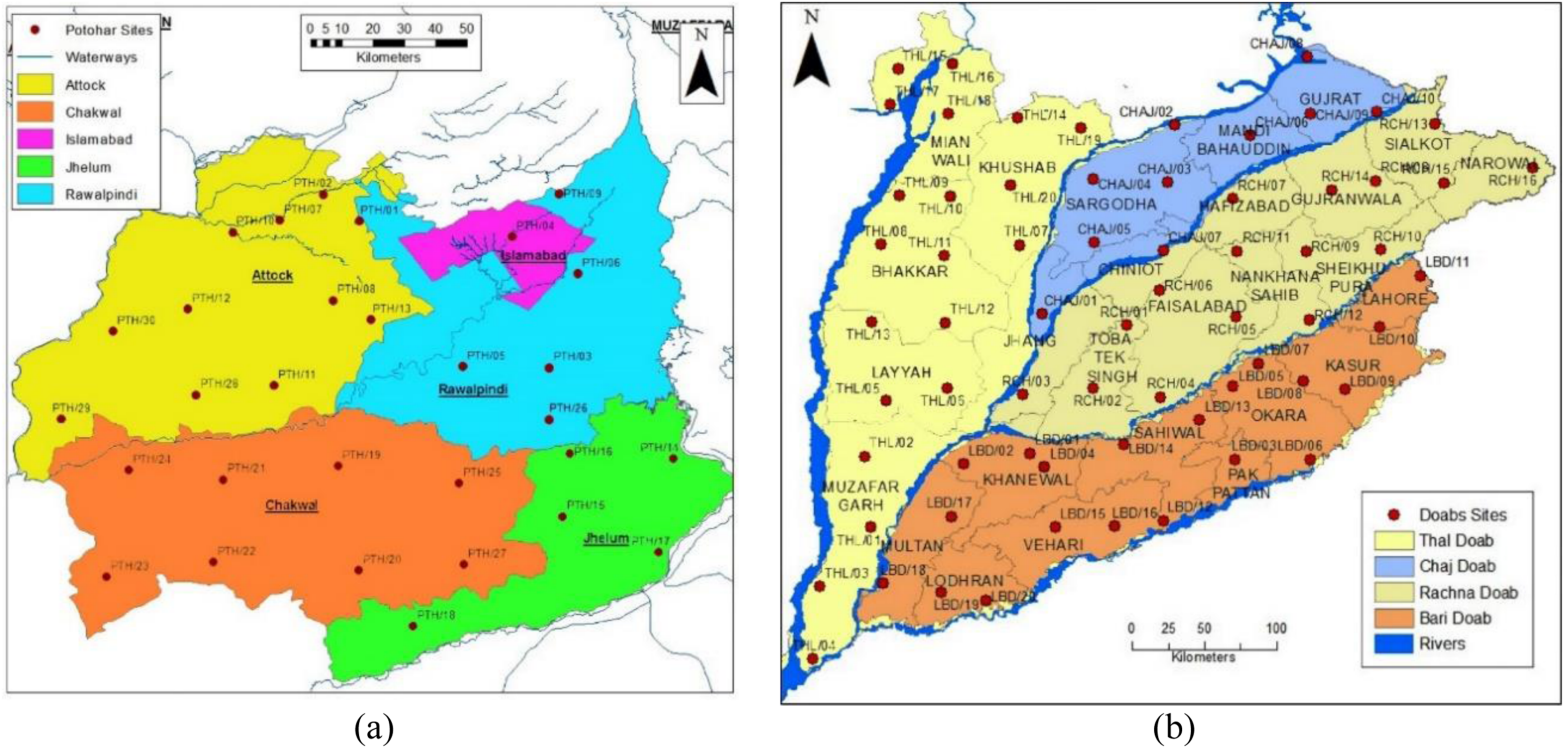
Geographical map of survey sites: (A) Pothwar; (B) Doabs. Figure source credit: © Malik et al. (2019).

The study area selected for this research is the Upper Indus Plain of Pakistan, having thirty sites in the Pothwar region and sixty-six in Doabs (the area between two rivers). These sites are taken from the research report, Soil Physical and Hydraulic of the Upper Indus Plain of Pakistan (Malik et al., 2019). The northeastern areas of Pakistan, including the districts of Jhelum, Chakwal, Rawalpindi, Islamabad and Attock, consist of 2.23 Mha called the Pothwar plateau, as shown in Fig. 1A. This plateau is geographically surrounded by a salt range in the south, River Indus and Jhelum in the west and east, respectively. The surface of this land has waves and dissected ditch belts. The dominant soil texture is silt loam, loam, and Sandy loam in Pothwar. The main cultivated crops of this area are barley, wheat, legumes, sorghum, and vegetables (Khan et al., 2018).

Doab is a land enclosed among two merging rivers. The most populous province of Pakistan, Punjab, has four doabs because it has five rivers streaming. These four Doabs are Thal, Rachna, Bari and Chaj Doab, as shown in Fig. 1B. Thal Doab is an area enclosed between Jhelum and Indus Rivers. It is the largest doab geographically, having area of 37,477 km^2^. Khushab, Mianwali, Layyah, Bhakkar, and Muzaffargarh are major districts of Thal Doab. Soil type of twenty sites were determined and each site covers 1,873 km^2^ area in Thal Doab (Liao et al., 2013). Rachna Doab is an area enclosed between Chenab and Ravi Rivers and is geographically the second largest doab having an area of 31,331 km^2^. It comprises the central districts of Punjab. Gujranwala, Sialkot, Narowal, Sheikhupura, Faisalabad, Nankana Sahib and Toba Tek Singh are major districts in Rachna Doab. Rachna Doab determined the soil type of sixteen sites; each covers a 1,958 km^2^ area. Bari Doab is an area enclosed between Sutlej, Bias and Ravi Rivers, having an area of 29,649 km^2^. Major districts of Bari Doab are Okara, Khanewal, Sahiwal, Multan, Lahore, and Kasur. The soil type of twenty sites was determined in this Doab; each site covers 1,482 km^2^ area. Chaj Doab is an area between Chenab and Jhelum Rivers and the geographically smallest Doab with an area of 13,660 km^2^. Sargodha, Mandi Bahauddin and Gujrat are major districts of Chaj Doab. The soil type of ten sites was determined in Chaj Doab; each site covers 1,366 km^2^ area.

**Figure 2:**
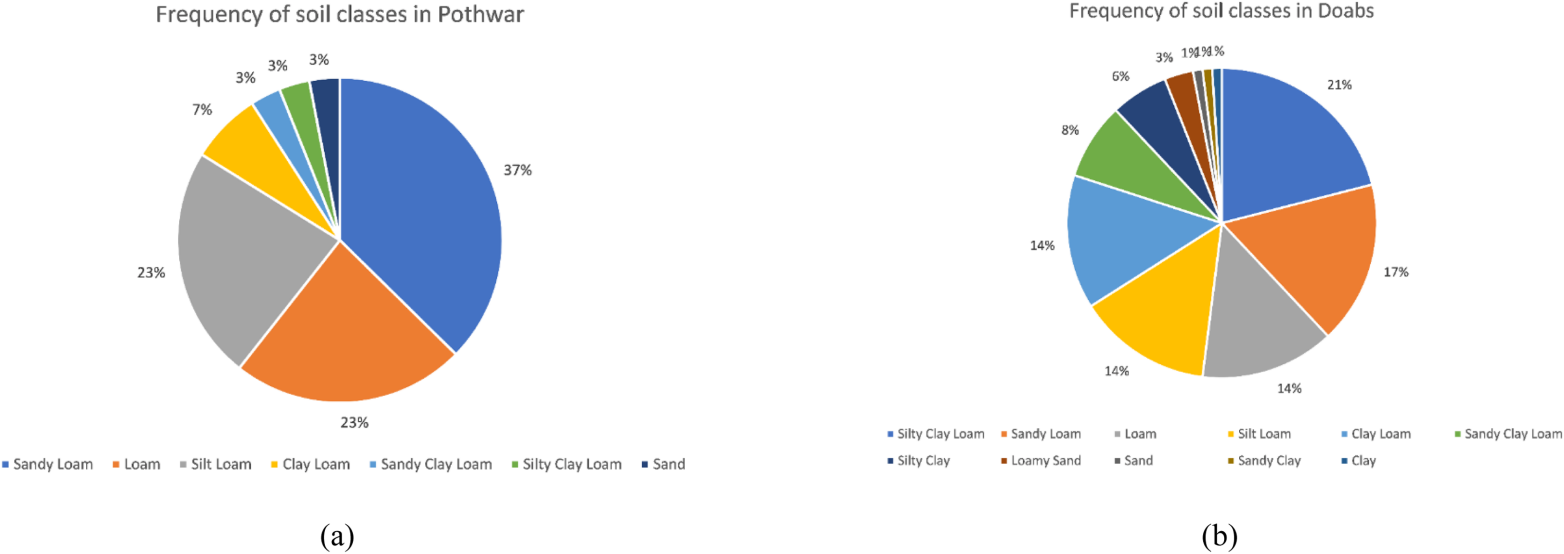
Frequency distribution of soil classes on surface: (A) Pothwar; (B) Doabs Full-size. Figure source credit: © Malik et al. (2019).

The Pits were denser in Pothwar than in Doabs because of the extra diversity in the geology of Pothwar. Pothwar has 22,254 km^2^ geographical area and 27.5 km × 27.5 km was the grid size. While 41.5 km × 41.5 km was the grid size of Doabs and 113,085 km^2^ was the geographical area (Khan et al., 2018). The distribution of soil classes in the surface of Pothwar and Doab regions is shown in Figs. 2A and 2B. Dominant soil classes of Pothwar plateau are sandy loam, loam, and silt loam. Similarly, dominant soil classes of Doabs are sandy loam, loam, and silt loam.

The soil type can be identified using the spectral signature of land with the help of remote sensing data. In this work, we used Landsat 8 data (Pahlevan et al., 2014) and supervised classification methods to achieve the results. Spectral images acquired through remote sensing satellites are used to identify suitable frequency bands to calculate spectral signatures.

### Satellite data

In 2013, the National Aeronautics and Space Administration (NASA) launched Landsat 8. It was a recently initiated Landsat satellite that housed the Operational Land Imager (OLI) and the Thermal Infrared Sensor (TIRS) instruments. OLI collects data for the visible, near-infrared, panchromatic band, and short-wave infrared spectral bands, whereas TIRS gathers images in the thermal region. The satellite completes its one cycle in 16 days and more than 1.8 million scenes are publicly available for the research community that can be utilized in several applications.

Fifteen cloud-free Landsat 8 images acquired from USGS Earth Explorer website (https://earthexplorer.usgs.gov) between June 2020 and August 2020 were used for the present study. Several preprocessing steps were performed on each image to get the spectral band values shown in Table 1. We calculated these spectral bands’ reflectance and corrective reflectance values. Soil signatures are commonly used in agricultural remote sensing and are known as vegetation indices. We also calculated the NDVI, EVI and SAVI vegetation indices (Rezaei et al., 2016) to investigate the impact of spectral bands on soil type classes. We have excluded Band 8–Panchromatic (0.50–0.68 µm) value due to the short range and most affected by clouds.

**Table 1:** Landsat 8 spectral bands characteristics.

Spectral bands	Wavelength (micrometers)	Spatial resolution (meters)
Band 1 (Coastal aerosol)	0.43–0.45	30
Band 2 (Blue)	0.45–0.51	30
Band 3 (Green)	0.53–0.59	30
Band 4 (Red)	0.64–0.67	30
Band 5 (Near Infrared (NIR))	0.85–0.88	30
Band 6 (SWIR 1)	1.57–1.65	30
Band 7 (SWIR 2)	2.11–2.29	30
Band 9 (Cirrus)	1.36–1.38	30

### Derived spectral band values

Spectral signature is a combination of spectral bands’ reflectance of material concerning wavelengths. It is used to help classify remote sensing images. We collected spectral band values, as shown in Table 1 for 82 out of 96 instances of the understudy site (Malik et al., 2019). We focused on five types of soil types: Silt Loam, Silty Clay Loam, Clay Loam, Sandy Loam and Loam which are dominant soil types in these regions, so we ignored fourteen instances that belonged to other soil types. On the base of Spectral band values, we have calculated reflectance band values, corrective reflectance band values and vegetation indices to generate our dataset. Our dataset has 27 attributes and a class label named soil type. We expanded our dataset by considering the nearest points and the 3 km distance from the actual points. Actual and extended instances of the dataset are given in Table 2. This labeled data is used to train a supervised machine learning algorithm that models the specific land map into different land types.

**Table 2:** Details of dataset.

Sr. No.	Soil type	Actual instances	Total instances
1	Silt loam	18	500
2	Silty clay loam	15	503
3	Clay loam	11	451
4	Sandy loam	22	505
5	Loam	16	504
Total		82	2,463

### Classifiers algorithms

Several classification methods have already been applied to measure soil type; among them, RF and SVM are commonly used. In this study, we have selected three multi-class classification methods to train and compare the soil type classification; more detail on these methods is given in the following subsection.

### Random forest

The random forest contains several decision trees. Each tree gives the class a vote, and the majority vote determines the final class. This implies that RF is an enhanced version of the decision tree classification. RS has also been extensively used in remote sensing classifications, where it has been applied to multitemporal images, multispectral data, optical data, and Synthetic Aperture Radar (Bousbih et al., 2019). In the most classical form of RF, a randomly built multiple decision tree is used, where each tree is trained on a random subset of data (named features). In recent decades, it has been known to be one of the most efficient algorithms and has been extensively reported in remote sensing literature. It has been widely used for various applications in the agricultural sector, including the analysis and classification of soil texture. The construction of classification and regression trees (CART) from the samples are involved in developing the RS classifier (Chen et al., 2017). A subset of input features (attributes) is randomly selected to form a set of predictor variables. It is best used for classification when there is a multi-class problem. The prediction of the RF depends on the predictions of the individual trees in the forest. The class that is classified by the random forest is the most predicted class in the individual trees. The important parameters that affect its performance: are the number of trees (k), maximum depth of the tree (not exceeding 25) and the minimum number of samples in each node (Bousbih et al., 2019). Typically, as the value of k increases, accuracy becomes better. Limiting the number of features reduces a RF’s computational complexity. In the present study, the parameters were set as shown in Table 3.

**Table 3:** Parameterization of random forest classifier.

Parameters	Configuration
Number of trees (k)	100
Maximum depth of the tree	19
Minimum number of samples in each node	1

### Support vector machine

The support vector machine (SVM) is a supervised algorithm, and it is used for the classification of both linear and nonlinear data. SVM is generally employed in classification, pattern recognition, and regression analysis (Bousbih et al., 2019). It has been frequently used in remote sensing classification applications (Mohamed et al., 2018). The classifier uses a nonlinear mapping to transform the original data into a higher dimension and essential training tuples called support vectors are used to find a hyperplane for the data separation. In the case of multi-class classification, the SVM classifiers use kernel function to the input data, where the data are not linearly separable. The polynomial kernel is used among the different kernel functions that have been already proposed. In the current study, the Polynomial kernel was picked. Therefore, any complex problem can be solved by choosing an appropriate kernel function in SVM.

### Logistic model tree

A logistic model tree (LMT) combines logistic regression (LR) and decision tree learning methods. In this model, information gain is used for splitting, the Logitboost algorithm is used to produce an LR model at every node in the tree and the tree is pruned using the CART algorithm (da Silva Chagas et al., 2016). LMT used cross-validation to find several Logitboost iterations to prevent training data overfitting. The Logitboost algorithm uses additive logistic regression, with each class Ci having a least square fit (Doetsch et al., 2009).

(1) $${{\rm L}_{\rm M}}\left( {\rm x} \right) = \mathop \sum \nolimits_{{\rm i} = 1}^{\rm n} \left( {{{\rm \beta }_{\rm i}}{{\rm x}_{\rm i}} + {{\rm \beta }_0}} \right)$$

where *n* is the number of factors, whereas β_i_ is the coefficient of the *i*th component of the vector x.

In the LMT model linear logistic regression method is used to calculate posterior probability in leaf nodes (Chen et al., 2017).

(2) $$P\left( {{\rm M|x}} \right) = \displaystyle{{\exp \left( {{{\rm L}_{\rm M}}\left( {\rm x} \right)} \right)} \over {\mathop \sum \nolimits_{{\rm {M}^{\prime}} = 0}^D {\rm exp}\left( {{{\rm L}_{{\rm {M}^{\prime}}}}\left( {\rm x} \right)} \right)}}$$

D is the number of classes.

### Evaluation

**Figure 3:**
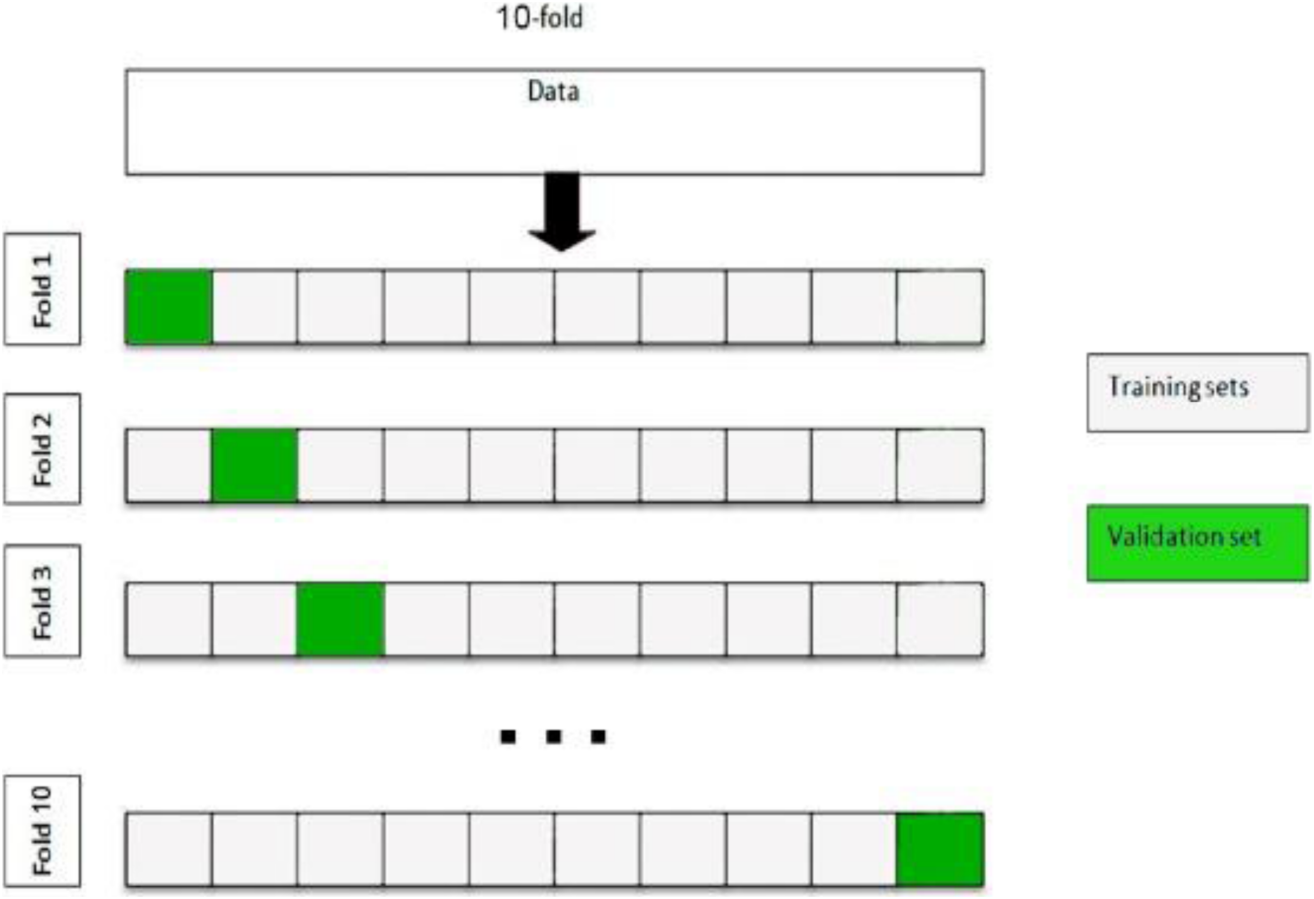
Ten-fold cross validation.

In this section, we compare the three approaches using their parameterization with three different classifiers (RF, SVM and LMT). The classification performance should be validated under conditions as close to the production environment. The results obtained are averaged over ten random, equally disseminated trails from the training and validation samples and not influenced by any specific split between the training and validation samples. For optimization of these classifiers, we used 10-fold cross-validation. The fundamental idea of this validation is to divide the training dataset into 10 subsets of equal size, with the ratios 9/10 and 1/10 being used for the training and validation sets, respectively. A classifier is trained on nine subsets and a remaining subset is used for validation. The split criteria of 10-fold cross-validation are shown in Fig. 3.

Validation is an essential step to evaluate the quality of supervised classification results and is achieved by evaluating the classified data with the reference data. The confusion matrix facilitates the understanding of results and provides detailed information concerning the effects of incorrect predictions. The confusion matrix is constructed on each experiment which exhibits the predicted and actual classes as shown in Table 4. The square array represents the confusion matrix where the column corresponds to the predicted values while rows represent the reference data. Matrix diagonal values represent the correctly classified values, whereas other elements correspond to confusion.

**Table 4:** Confusion matrix.

Confusion matrix		Actual class	
		Yes	No
Predicted class	Yes	TP	FP
	No	FN	TN

(3) $${\rm Accuracy} = \displaystyle{{TP + TN} \over {TP + FP + TN + FN}}$$

(4) $${\rm Precision =}\displaystyle{{{\rm TP}} \over {{\rm TP} + {\rm FP}}}$$

(5) $${\rm Recall =}\displaystyle{{{\rm TP}} \over {{\rm TP} + {\rm FN}}}$$

(6) $${\rm F1 Score =}\;2*\displaystyle{{\left( {{\rm Recall*Precision}} \right)} \over {\left( {{\rm Recall} + {\rm Precision}} \right)}}$$

To evaluate the influence of the class label, using RF, SVM and LMT, the Accuracy, Precision, Recall and F1 Score are computed from each confusion matrix over a set of different simulations.

## Results

### Satellite data sensitivity to soil type

#### Landsat-8 sensitivity analysis

**Figure 4:**
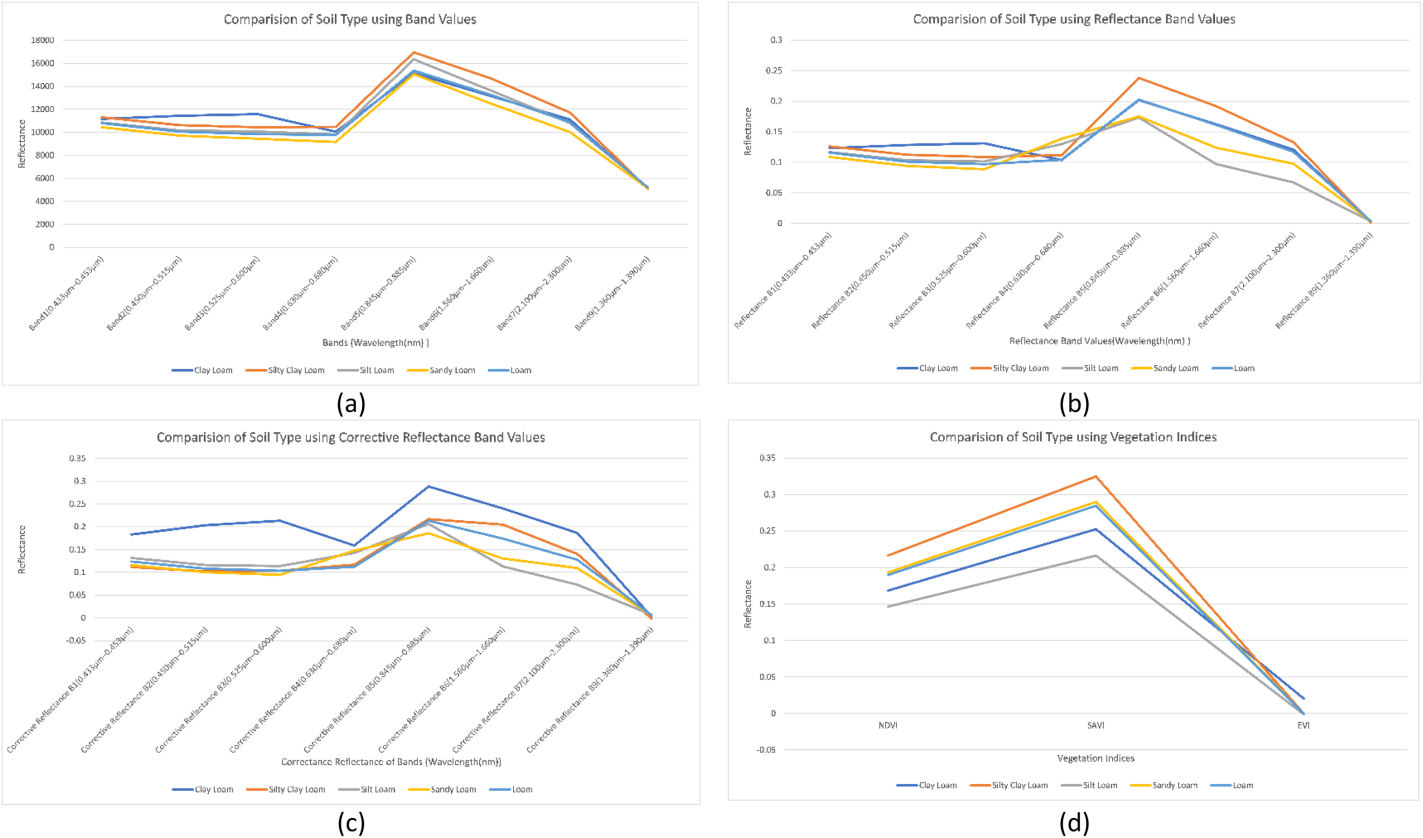
Comparison of soil types: (A) using mean of band values; (B) using mean of reflectance band values; (C) using mean of corrective reflectance band value*s*; (D) using vegetation indices.

We analyzed the impact of band values, reflectance band values, corrective reflectance band values and vegetation indices on soil type classes. We found that the reflectance of Band 1, Band 4 and Band 9 almost remains the same for all types of soil type classes. Furthermore, Band 2, Band 3, Band 5, Band 6 and Band 7 reflectance are more sensitive toward soil type, as shown in Fig. 4A. Reflectance band values of Band 5, Band 6 and Band 7 efficiently differentiate the soil types. Spectral signatures of reflectance band values helped us to map the soil type as shown in Fig. 4B. Clay loam has the highest reflectance values for corrective reflectance bands from Band 1 to Band 7. Corrective reflectance values for Band 6 and Band 7 have a significant impact on soil type classes, as shown in Fig. 4C. Silt Loam has minimum reflectance. However, Silty clay loam has the highest reflectance on NDVI and SAVI vegetation indices. From NDVI, SAVI and EVI vegetation indices, NDVI and SAVI can be seen to have sensitivity to soil types, as shown in Fig. 4D.

#### Classification scheme

**Figure 5:**
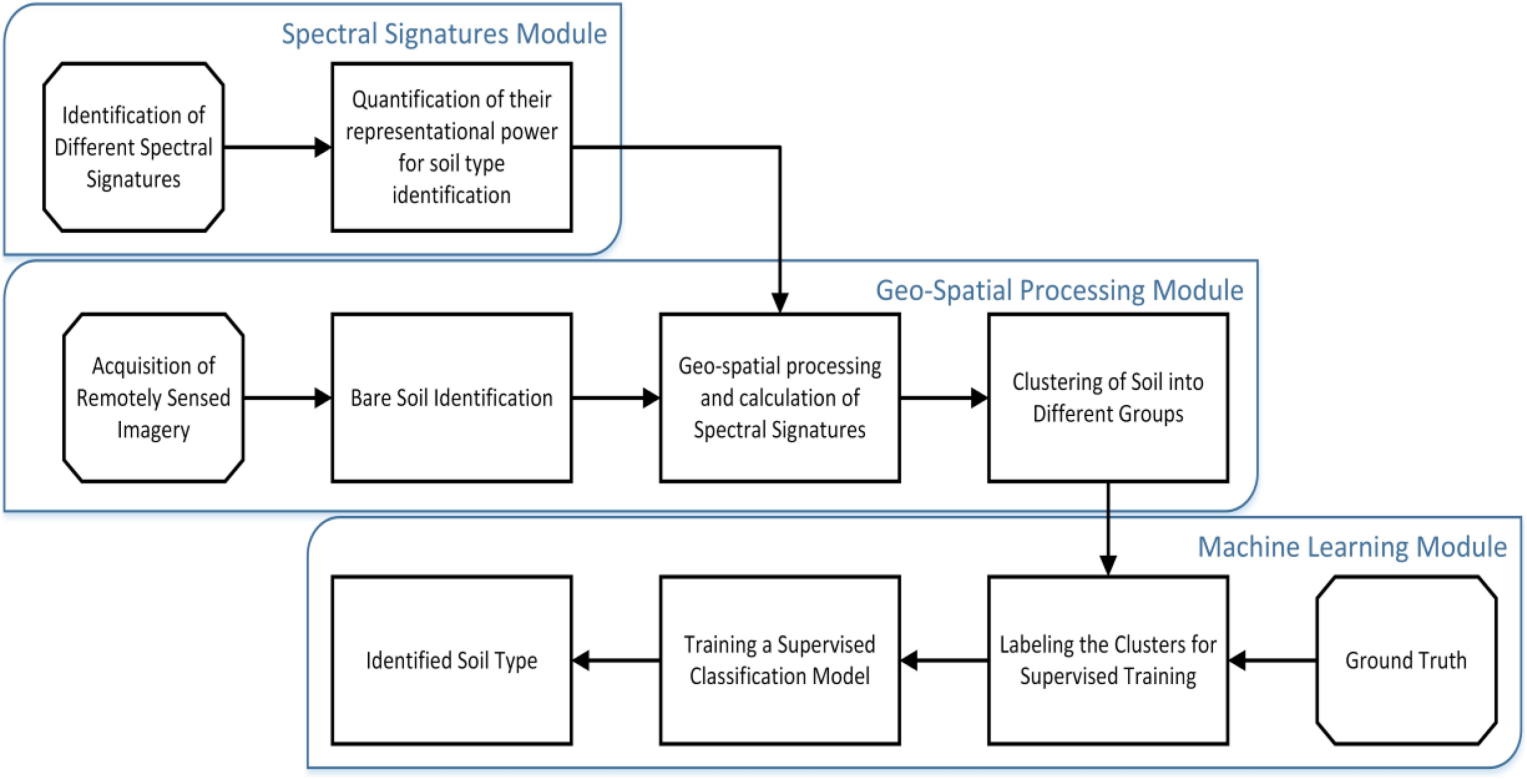
Classification workflow of soil type identification.

Twenty-seven indicators were used to estimate the soil type: Landsat 8 images in eight different bands (B1–B7 and B9), the reflectance of band values, corrective reflectance of band values and vegetation indices. From sensitivity analysis, we found that all these indicators are sensitive to soil type class, as shown in Fig. 4. Consequently, all spectral bands were selected for the classification process. The workflow of the present study with its three modules to perform the soil type identification using remote sensing data is shown in Fig. 5.

#### Validation

**Figure 6:**
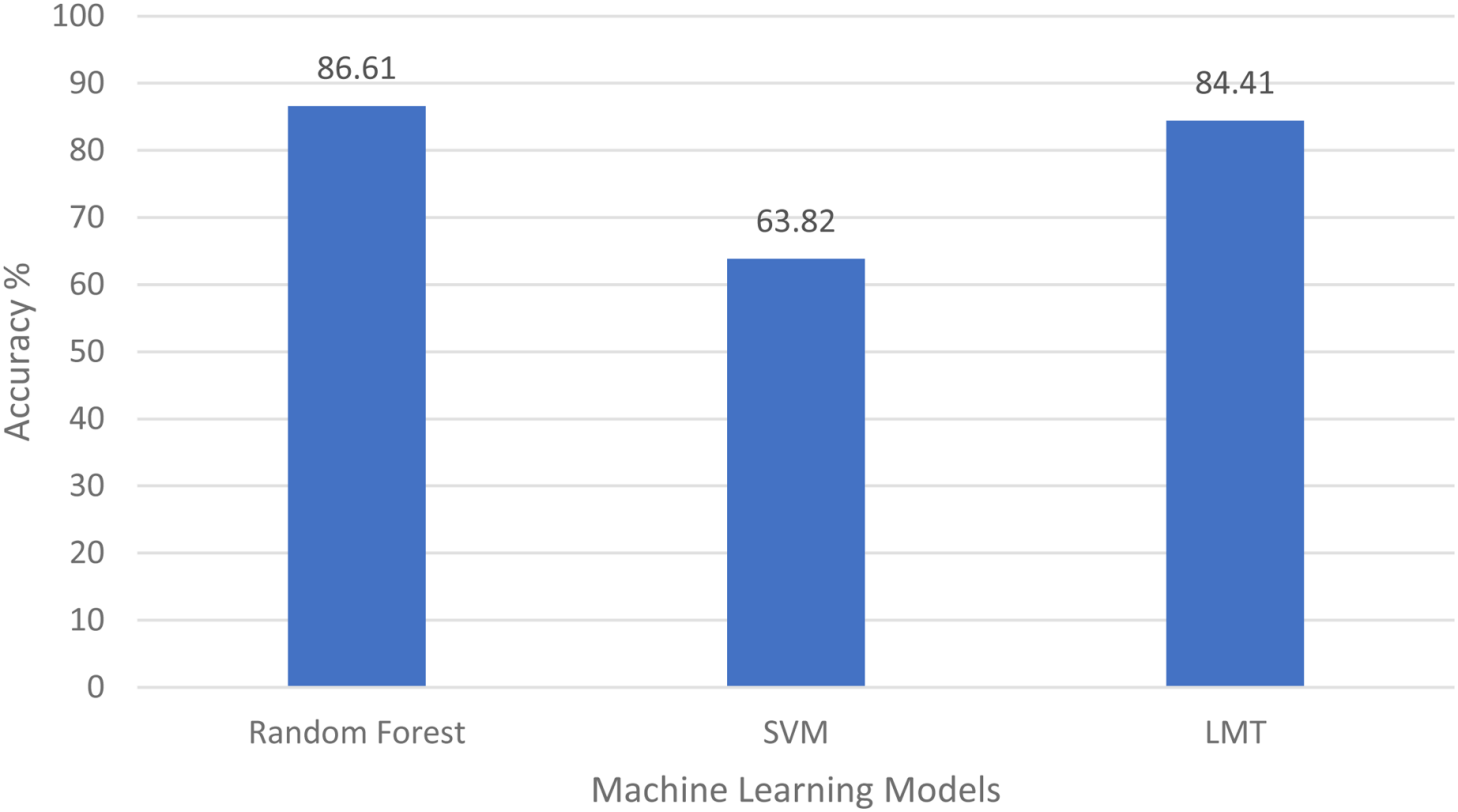
Comparison of the algorithm’s accuracies.

A 10-fold cross-validation procedure is used to obtain predictions from the different classifiers. The accuracies obtained from the confusion matrices with these simulations are given in Fig. 6. It was difficult for classifiers to differentiate Clay Loam from Silty Clay Loam and Sandy Loam from Silt Loam. The highest accuracy, 86.61% is obtained while identifying the soil type with the RF algorithm. The results obtained with Landsat 8 spectral data show that the RF is a more robust classifier than others for all the experiments carried out.

**Figure 7:**
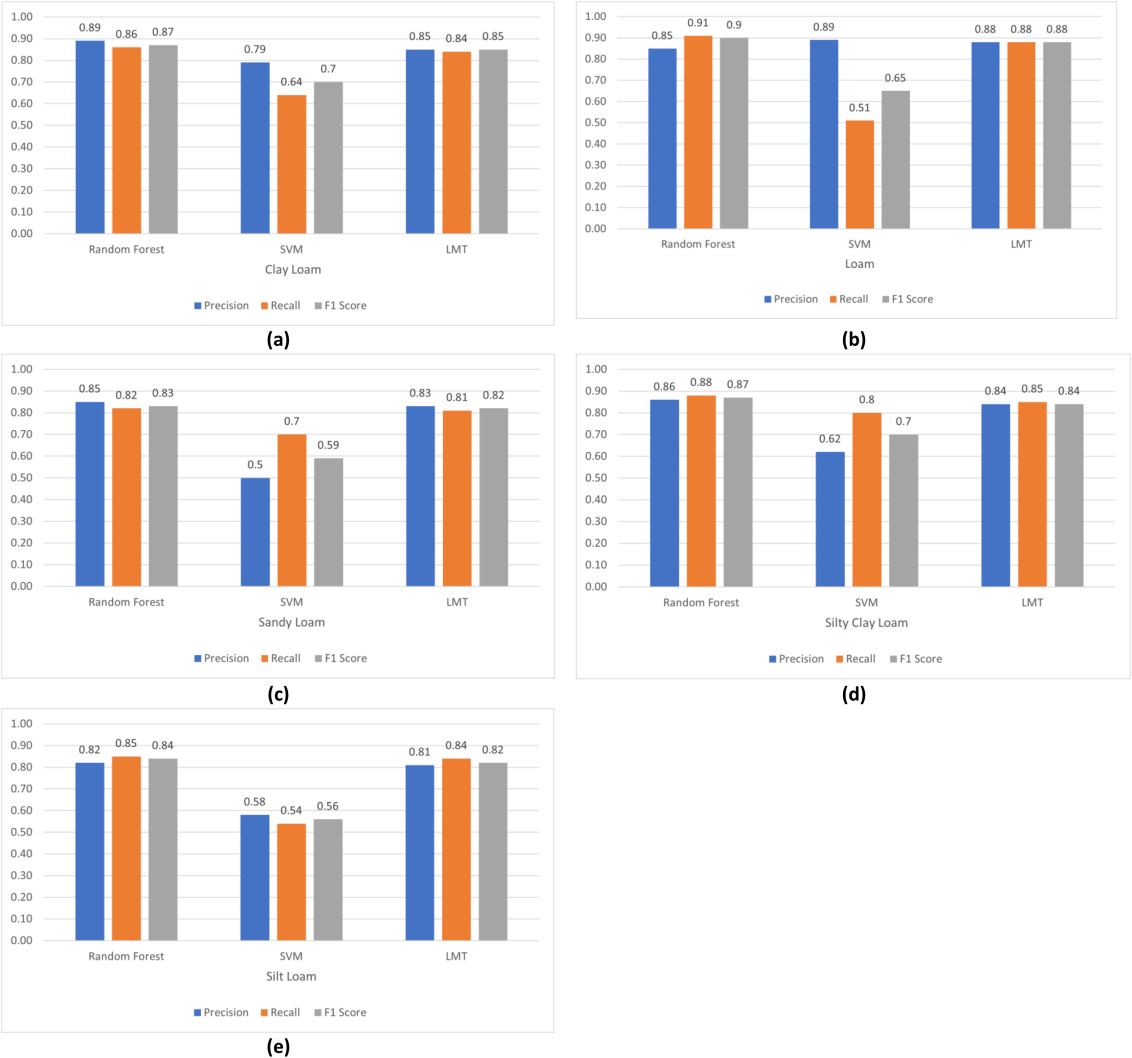
Comparison of algorithm’s precision, recall and F1 score: (A) clay loam; (B) loam; (C) sandy loam; (D) silty clay loam; (E) silt loam.

The comparison of Precision, Recall and F1 Score of these models for loam, sandy loam, clay loam, silty clay loam and silt loam are elaborated in Figs. 7A–7E.

## Discussion

The first research problem was “Is it possible to identify the type of soil using remote sensing data?” We have identified the soil type by using spectral signatures using remote sensing data. The different bands values of the pixel differentiate the soil type as shown in Figs. 4A–4D. Results also show us that, soil types are identified using remote sensing data with 86.61% accuracy by applying a random forest machine learning model as shown in Fig. 6. In the identification of soil type, clay loam was misclassified with silt clay loam and loam, sandy loam with silt loam and loam, silty clay loam with clay loam and silt loam, loam with sandy loam and silt loam, silt loam with sandy loam and loam.

Our second research question was “How can we map spectral signature with soil type?” For this, we have studied the impact of band values as in Fig. 4A, reflectance values shown in Fig. 4B, corrective reflectance values shown in Fig. 4C and vegetation indices shown in Fig. 4D on soil type. We observed that for clay loam Band 2, Band 3, reflectance of Band 2, Reflectance of Band 3, corrective reflectance of Band 1 to corrective reflectance Band 8 and NDVI and SAVI are the spectral signatures which more helpful to differentiate it from another soil type. For silty clay loam spectral signature of Band 5, reflectance of Band 5, reflectance of Band 6, reflectance of Band 7, corrective reflectance of Band 6, corrective reflectance of Band 7, NDVI and SAVI are used to differentiate it. More helpful spectral signatures for silt loam are Band 5, Band 6, reflectance of Band 6, reflectance of Band 7, corrective reflectance of Band 6, corrective reflectance of Band 7, NDVI and SAVI. The spectral signatures of Band 4, Band 5, Band 6, Band 7, reflectance of Band 3, reflectance of Band 6, reflectance of Band 7, corrective reflectance of Band 5, corrective reflectance of Band 6, corrective reflectance of Band 7, NDVI and SAVI. Similarly, more useful spectral signatures for loam are Band 5, reflectance of Band 7, corrective reflectance of Band 6, corrective reflectance of Band 7. The spectral signatures of loam and sandy loam is very close for Band 5, NDVI and SAVI. Overall Band 5, reflectance of Band 5, reflectance of Band 6, corrective reflectance of Band 5, corrective reflectance of Band 6, NDVI and SAVI are more valuable spectral signatures.

Our third research question was “Which index calculated from remotely sensed images is more useful for soil type identification?” Based on our research work NDVI and SAVI are more useful indices among NDVI, SAVI and EVI vegetation indices as we discussed in this work. NDVI and SAVI index values are more helpful to identify the soil type as shown in Fig. 4D.

Fourth and last research question was “Which machine learning algorithm is more effective at mapping the spectral signature to relevant soil type?” For this, we have implemented different machine learning techniques like Random Forest, support vector Machine, decision trees, logistic model tree and bagging to identify the soil type. Accuracies of these approaches are elaborated in “Discussion”; you can clearly see the Random Forest, which is an ensemble learning algorithm, gave us the highest accuracy 86.61% as shown in Fig. 6.

## Conclusion

Soil type plays an important role in the cultivation of the crop in a specific area. In the present study, we identified the soil type using spectral signatures based on remote sensing data. We also analyzed the impact of band values, reflectance values, corrective reflectance values, and vegetation indices on soil type. Band 5, reflectance of Band 5, reflectance of Band 6, corrective reflectance of Band 5, and corrective reflectance of Band 6 are more sensitive for soil type classes. Spectral signatures of NDVI and SAVI are more helpful in identifying the soil type. Three different machine learning techniques, Random Forest, support vector machine and logistic model tree, have been used to identify soil type classes and achieved 86.61% accuracy using Random Forest. Government organizations and decision-makers can use these results for agricultural planning. In future work, we can increase the number of soil type classes to map more areas. Consequently, the web-based application can be designed to determine the soil type by giving the location. Many machine learning classification techniques can be used to improve this multi-class classification problem. For example, we can use neural networks as deep learning algorithms to get better results from this dataset.

## Supplemental Information

10.7717/peerj-cs.1109/supp-1 Supplemental Information 1 instructions to configure code.

10.7717/peerj-cs.1109/supp-2 Supplemental Information 2 Dataset.

## Additional Information and Declarations

### Data Availability

The following information was supplied regarding data availability: The code and data are available in the Supplemental Files.
