# Supplementary material for: Identification of soil type in Pakistan using remote sensing and machine learning
Source: PeerJ Comput Sci. 2022 Oct 3;8:e1109. doi: 10.7717/peerj-cs.1109 (PMC9575843; doi:10.7717/peerj-cs.1109)
Supplement: Supplemental Information 1 [file peerj-cs-08-1109-s001.docx]

The following machine learning algorithms were used to get the results for this study in Weka software.

1. Random Forest
2. Support Vector Machine
3. Logistic Model Tree

Reader can get the results by following these steps.

Steps:

1. Open weka software or download it from internet using this link <https://waikato.github.io/weka-wiki/downloading_weka/>.
2. Click on explorer button using Weka.
3. Then press open file button to choose the dataset (Soil_Type_Dataset).
4. After that press on classify option given in toolbar.
5. Choose Random Forest as classifier with the following parameter settings.
   1. BagSizePercent=100,batchsize=100,maxDepth=19,numDecimalPlaces=2,numExecutionSlots=1,numFeatures=0;numIteration=100,seed=1.
   2. All remaining parameters are set to be false.
6. Choose SMO (for SVM implementation in weka) as classifier with the following parameter settings.
   1. batchsize=100,c=1,calibrator=Logistic,epsilon=1.0E12,numDecimalPlaces=2,kernel=polykernel,numFeatures=0;numIteration=100,numFolds=1,randomSeed=1,toleranceParameter=0.001.
   2. All remaining parameters are set to be false.
7. Choose LMT as classifier with the following parameter settings.
   1. batchsize=100,minNuminstaces=15,numBoostingIterations=-1, numDecimalPlaces=2,weightTrimBeta=0.0,
   2. All remaining parameters are set to be false.
